# Development of an Analytical Model to Describe the Disperse Melting in Wave-Dispersion Screws

**DOI:** 10.3390/polym12040946

**Published:** 2020-04-18

**Authors:** Marius Dörner, Christian Marschik, Volker Schöppner, Georg Steinbichler

**Affiliations:** 1 Kunststofftechnik Paderborn, Paderborn University, 33098 Paderborn, Germany; volker.schoeppner@ktp.uni-paderborn.de; 2 Institute of Polymer Extrusion and Compounding, Johannes Kepler University Linz, 4040 Linz, Austria; christian.marschik@jku.at (C.M.); georg.steinbichler@jku.at (G.S.)

**Keywords:** wave-dispersion screw, modeling and simulation, polymer processing, extrusion, melting

## Abstract

The progressive development of new screw concepts in single screw extrusion also makes it necessary to develop new models for the correct process description. When looking at wave-dispersion screws, the disperse melting behavior should be mentioned in particular, which has so far been less researched and modeled than the conventional melting behavior, as it occurs in standard screws. Therefore, an analytical model is presented in this paper, which considers the disperse melting under consideration of the melt and solid temperature. The basic assumption is Fourier heat conduction from the melt surrounding the particles into the particles. Furthermore, the melt temperature development by dissipation and the cooling effects were modeled analytically. Additionally, the solid bed temperature was modeled by a 2D-FDM method. By dividing the screw into several calculation sections with constant boundary conditions, it was subsequently possible to calculate the melting process over the screw length. The model developed shows comprehensible results in verification and successfully reproduces the solids content over the screw length with a mean deviation of absolute 11% in validation tests using cooling/pulling-out experiments.

## 1. Introduction

In the plastics processing industry, the focus is more and more on optimizing the efficiency of extrusion lines. Particularly in single-screw extrusion, the quantitatively dominant process in plastics processing, this is achieved by raising the screw speed while keeping the machine size constant, thereby increasing throughput. However, this is not possible indefinitely, as it often leads to unacceptable plastication and homogenization of the polymer. In order to ensure a sufficiently high melt quality at higher speeds, new screw concepts are being developed in addition to conventional screws, which have not yet been fully researched. Particularly noteworthy are the wave-dispersion screws, which promise increased throughput while maintaining the same melt quality. By breaking up the solid bed at an early stage in the process, they initiate a disperse melting process, which is intended to optimize the melting and temperature behavior. These include, for example, double-wave and energy-transfer screws, which are explained in the following.

Wave-dispersion screws, which were first patented by Kruder in 1972 [1], are characterized by the periodic increase and decrease of the channel depth in the metering section. When unwound, this results in a wave-shaped screw channel through which the material is conveyed. At the wave crests, the points of minimum channel depth, the material is subjected to increased shear [1]. In addition, the elongation flows introduced by the channel shape cause the solid bed to break up, whereby the individual solid particles are distributed in the already formed melt matrix. Due to the large surface area of the distributed granulate particles in the melt, a more effective melting can take place. As a result, the melting performance is increased, while the melt temperature is expected to drop [2]. All in all, higher speeds with better melt quality can be achieved than with conventional screws [1]. A disadvantage, however, is that an incorrectly designed geometry can lead to plugging of the extruder and thus to pulsating or reduced output [3]. For this reason, the double-wave (DW) and energy-transfer (ET) screws were developed.

**Figure 1:**
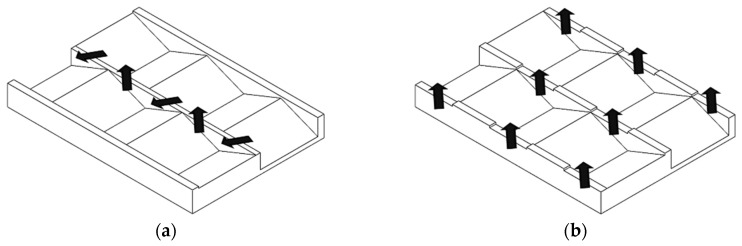
Unwound channel of a double-wave screw (**a**) and energy-transfer screw (**b**).

The double-wave screw was patented by Kruder in 1978 [4]. It maintains the periodic increase and decrease of the flight depth, but divided into two channels (see Figure 1). The waves are arranged offset, so that one channel has a wave trough while the other channel has a wave crest and vice versa [5]. Similar to a barrier screw, the two channels are separated by a continuous secondary flight, which has a larger gap to the barrel than the main flight [4]. This solves the problem of clogging of the wave-dispersion screw, since both melt and solids are moved to the other channel via the offset flight. In addition, by splitting the melt at a wave crest via the secondary flight and the wave crest itself, mixing is strongly promoted, and thus a higher thermal homogeneity is achieved [4]. This also promotes the mixing of the individual solid particles with the melt, so that a high plasticizing capacity is achieved with low heat input into the melt [3,6,7]. The design of double-wave screws is difficult. Several investigations have shown that the wave geometry has a very large influence on the behavior of the screw. Therefore, the design must be systematically and well elaborated in order to use the full potential of the double-wave concept [3].

The energy-transfer screw (ET Screw) was developed by Chung and Barr [8]. Its structure is similar to that of the double-wave screw. However, the main and secondary flights are separated so that the melt can flow over the flight into the upstream screw channel at each wave crest (see Figure 1). The functions of the main and secondary flights alternate in such a way that the melt repeatedly changes channels against the direction of the flow [3]. This is a significant difference to the double-wave screw, where the melt flows partly in and partly against the conveying direction due to the fact that only the secondary flight is set off. This flow pattern leads to an increased mixing effect of the screw which is supposed to greatly improve the melting behavior. As with the double-wave screws, this results in a higher possible throughput at lower melt temperatures. These properties have been investigated and verified in several experimental studies [5,9,10,11,12,13]. The complex screw geometry, however, requires a sophisticated design and manufacture to achieve the desired flow behavior of the screw.

In order to enable a correct design of the screw geometries for the wave-dispersion screws mentioned above, accurate modeling is of the highest relevance. In this paper an analytical model is presented which allowed us to calculate the melting behavior of the screws to simplify the design. In the following, the melting models will be discussed. Another important process variable is the pressure-throughput behavior of the wave-dispersion screws. Here, we refer to the publications [14,15].

## 2. Melting Models

**Figure 2:**
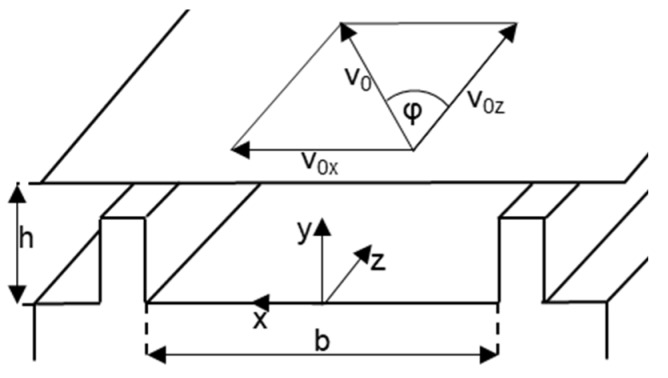
Kinematic reversal.

One of the main tasks of the plasticating extruder is to melt the polymer granulate. For a satisfactory melt quality, no solid particles must be present in the melt when it leaves the forming die. Therefore, a reliable description of the melting process is a necessary condition to ensure a successful process design. This section, therefore, deals with the two main models used to model the melting process: conventional and disperse melting. Both models use kinematic reversal, as described in [16] (see Figure 2). Here, the screw rotation is not considered to be kinematic, but the barrel is moved over the screw accordingly. This creates a corresponding relative system. For further simplification, the screw channel is not wound, but unwound. It lies flat in the plane, while the barrel wall moves over this plane. The speed components for the barrel wall result from the peripheral speed of the screw v_0_ and the pitch angle φ.

(1) $$v_{0}=\pi \cdot N\cdot D$$

(2) $$v_{0z}=v_{0}\cdot \cos\left(\varphi \right)$$

(3) $$v_{0x}=v_{0}\cdot \sin\left(\varphi \right)$$

### 2.1. Conventional Melting Model 

**Figure 3:**
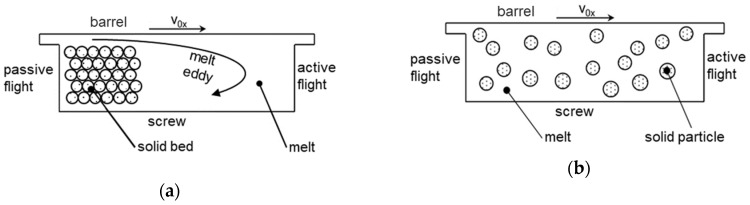
Schematic representation of the conventional (**a**) and disperse (**b**) melting models.

Typical models of conventional melting include the model of Tadmor [17], which was published as early as 1966, or the compact melting model according to Potente [16]. These conventional melting models assume the solid bed as a compact block. In the beginning, this fills the entire screw channel. Before the actual melting process starts, a melt film begins to form between the solids bed and the barrel wall. As soon as this melt film is sufficiently thick, there is a transverse flow in the melt channel, causing the melt to attach itself to the active flight of the channel in a melt eddy. This is done by scraping of the melt film on the barrel by the flight. Through the melt eddy, the solid bed is pressed against the passive flight and thus maintains its shape (see Figure 3). The further the melting process progresses, the smaller the solids bed and the larger the melt eddy becomes. The thickness of the melt film remains constant because the solid bed is further stabilized by the melt eddy. Furthermore, dissipative effects and heat conduction from the barrel wall lead to a temperature development in the melt eddy. The melting process continues until the entire material has melted [7].

### 2.2. Disperse Melting Model

Disperse melting is fundamentally different from conventional melting. Here, the starting point is not a compact solid bed, but an ideal distribution of the solid particles in the melt. Therefore, there is no clear distinction between the solid bed and the melt eddy. Furthermore, it is assumed that the solid particles are all spherical and are of identical size. Melting in this model is based on the assumption of a heat flow of the melt surrounding the particles into the melt itself. Due to the distribution of the particles within the melt, this melting model is particularly suitable for describing the melting process in screws which break up the solid bed. In this context, the double-wave or the energy-transfer screw should be mentioned in particular. However, screws with mixing elements at an early stage or high-speed extrusion can also lead to disperse melting.

The first mathematical description of disperse melting for single-screw extruders goes back to Huang and Peng [18,19,20]. Their model, also known as the six-block model, is based on a division of the channel cross-section into several blocks in which the melt and solid material are separate. Differential equations can then be formulated for the different blocks, which are dependent on each other. However, it should be critically considered that the completely numerical approach is based on many simplifications, so that, for example, convection within the melt is neglected.

A more recent model to describe disperse melting in single-screw extruders is provided by Rauwendaal [21]. Therein, the granulate is assumed to be ideally distributed in the melt. In addition, it is assumed that the shear-induced dissipation is balanced by the required melting energy. The additional assumption that there is no temperature change within the solid particle results in very short melting lengths in the calculation [3,22]. However, the Rauwendaal model only considers the heat flow through heat conduction into the particle. Chung and Barr [23] have already shown in a series of investigations in 1990 that the influence of convection on the melting calculation has to be taken into account for accurate analyses. They investigated the melting behavior of solid particles in silicone oil. It could be shown that the temperature in the particle center is increased and that the flow condition of the surrounding liquid also has a great influence on the melting time. Therefore, in addition to pure heat conduction, the influence of convection should also be considered in models of disperse melting. This has been taken into account by Pape [3], who considered the influence of convection and the temperature profile in the particle in his disperse melting model.

The described models all neglect essential aspects which significantly influence the disperse melting process. For example, the fundamentally different temperature calculation of the melt surrounding the particles must be taken into account. The homogeneous distribution of the particles causes them to cool down. Furthermore, the particles lead to an increase in shear in the melt, which leads to an increased dissipative energy input. Since the disperse melting is significantly influenced by the surrounding melt temperature, these two effects cannot be neglected. Furthermore, the initial temperature of the granulate at the beginning of the disperse melting is extremely relevant. The disperse melting usually starts after a certain residence time in the extruder, which is why the plastic granulate already reaches a higher temperature than the input temperature. This energy difference no longer needs to be transferred from the melt to the particle, which melts faster. In addition to a general modeling of the disperse melting process, these aspects are also included in the model described in this work.

## 3. Mathematical Treatment of Existing Melting Models

In order to be able to correctly describe disperse melting, conventional melting and its modeling must first be briefly discussed. As the disperse melting is usually always preceded by a conventional melting, this is essential. Furthermore, the basic of the disperse melting model is shown.

### 3.1. Conventional Melting Model

**Figure 4:**
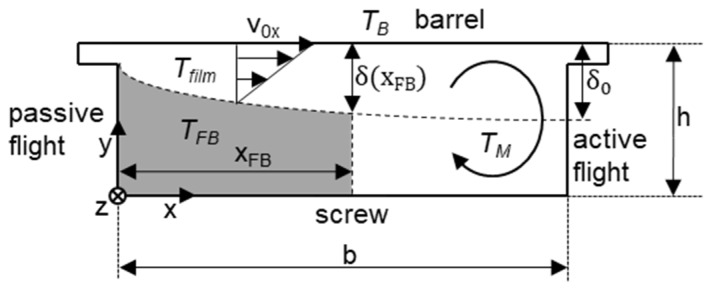
Schematic representation of conventional melting based on Potente [16].

The mathematical treatment of conventional melting is based on the model of Potente [16]. For this purpose the melt channel is divided into solid bed, melt film and melt eddy (see Figure 4).

First, the contour of the melt film is determined. This can be described by the following equation:

(4) $$\delta \left(x\right)=\delta_{0}\cdot {\left(\frac{x}{b}\right)}^{c}$$

Here, δ(x) describes the thickness of the melt film at position x, δ_0_ the thickness of the melt film at position x = b and b the channel width. The contour exponent c is determined iteratively via material and process data. For this purpose, two pairs of values of the thickness of the melt film δ(x_FB_) and the normalized solid bed width x_FB_/b are used. Here, T_FL_ is the melting temperature of the polymer; the explanation of the other individual factors used can be found in the nomenclature.

(5) $$\frac{x_{\mathrm{FB}}}{b}=\frac{k_{1}\cdot \delta {\left(x_{\mathrm{FB}}\right)}^{2}\cdot \left(1-c\cdot n\right)\cdot v_{0x}\cdot \rho_{m}\left(T_{M}\right)\cdot \left({\Delta h}_{m}+{\Delta h}_{s}\right)}{\left(2\cdot \frac{1-\mathrm{cn}}{1-c}+\frac{k_{2}\cdot K\left(T_{\mathrm{FL}}\right)\cdot v_{\mathrm{rel}}^{2}}{\lambda_{m}\left(T_{M}\right)\cdot \left(T_{B}-T_{\mathrm{FL}}\right)}\cdot {\left(\frac{v_{\mathrm{rel}}}{\delta \left(x_{\mathrm{FB}}\right)}\right)}^{n-1}\right)}\cdot \frac{1}{\lambda_{m}\left(T_{M}\right)\cdot \left(T_{B}-T_{\mathrm{FL}}\right)\cdot b}$$

(6) c=log(δ(xFB1)/δ(xFB2))log((xFB1/b)(xFB2/b))

(7) $$A=\frac{\beta }{n}\cdot \left(T_{B}-T_{\mathrm{FL}}\right)$$

(8) $$k_{1}=2\cdot \left(\frac{1}{1-e^{A}}+\frac{1}{A}\right)$$

(9) $$k_{2}=\frac{2}{A^{2}}\cdot {\left(\frac{A}{e^{A}-1}\right)}^{1+n}\cdot \left(e^{A}-A-1\right)$$

After determining the contour exponent c, the melt film thickness δ_0_ can be determined according to Equation (10). The width of the solid bed x can subsequently be calculated iteratively using Equation (11). For this purpose, an area balance of the area of the melt eddy, of the melt film and of the solids bed is performed for a given solids volume fraction ψ_v_:

(10) $$\delta_{0}^{2}=\left(2\cdot \left(\frac{1-c\cdot n}{1-c}\right)+\left(\left(\frac{k_{2}\cdot K\left(T_{\mathrm{FL}}\right)\cdot \left(v_{\mathrm{rel}}^{2}\right)}{\lambda_{m}\left(T_{M}\right)\cdot \left(T_{B}-T_{\mathrm{FL}}\right)}\right)\cdot \left({\left(\frac{v_{\mathrm{rel}}}{\delta_{0}}\right)}^{n-1}\right)\right)\right)\cdot \left(\frac{\lambda_{m}\left(T_{M}\right)\cdot \left(T_{B}-T_{\mathrm{FL}}\right)\cdot b}{k_{1}\cdot \left(1-\left(c\cdot n\right)\right)\cdot v_{0x}\cdot \rho_{m}\left(T_{M}\right)\cdot \left({\Delta h}_{m}+{\Delta h}_{s}\right)}\right)$$

(11) $$\frac{A_{\mathrm{solid}}}{A_{\mathrm{channel}}}=1-\frac{A_{\mathrm{meltfilm}}}{A_{\mathrm{channel}}}-\frac{A_{\mathrm{melteddy}}}{A_{\mathrm{channel}}}=1-\frac{\int_{0}^{x_{\mathrm{FB}}}\delta_{0}\cdot {\left(\frac{x}{b}\right)}^{c}\mathrm{dx}}{b\cdot h}-\frac{\left(b-x_{\mathrm{FB}}\right)\cdot h}{b\cdot h}=\psi_{v}$$

The mean temperature in the melt film can be described with the boundary conditions that the temperature of the melt assumes the barrel temperature T_B_ at the barrel wall and the melting temperature T_FL_ at the edge of the solid bed, by means of the following mathematical relationship according to [24]:

(12) $$T_{\mathrm{film}}=T_{\mathrm{FL}}+\left(T_{B}-T_{\mathrm{FL}}\right)\cdot \frac{\left(\left(\frac{1}{A}\right)-\left(\frac{A}{2}\right)+e^{A}\cdot \left(1-\left(\frac{1}{A}\right)\right)\right)}{e^{A}-A-1}$$

The melting process can now be represented by a melting mass flow ṁ_m_ as a function of the channel length z. This is based on the assumption that the melt film passes into the melt eddy at position x with a classical velocity profile of a drag flow, which results from the velocity transverse to the channel v_0x_ (see Figure 4). Due to the triangular shape of this profile, the mean velocity profile can be assumed to be 0.5∙v_0x_. Furthermore, the correction factor k_1_ must be taken into account when considering the non-Newtonian flow behavior. This results in Equation (13).

(13) $$\frac{\Delta {\dot{m}}_{m}}{\Delta z}=0.5\cdot v_{0x}\cdot \delta \left(x_{\mathrm{FB}}\right)\cdot \rho_{m}\left(T_{\mathrm{film}}\right)\cdot k_{1}$$

To describe the changing melting capacity over the entire unwound screw length z, an iterative procedure must be chosen. For that purpose, the screw is divided into many individual sections in which the melting capacity and the resulting solid volume fraction ψ_v_ can be determined. Thus, a melting process can be calculated over the screw length. This is needed to calculate the proportion of melt at the start of disperse melting.

Furthermore, it is relevant to know the temperature of the melt at the beginning of the disperse melting process, as this temperature has a significant influence on the disperse melting process. In the case of conventional melting, the model of Lakemeyer [25] can be used. This uses the dimensionless key figures Graetz (Gz) and Brinkmann (Br) to determine the temperature above the channel height in the melt eddy. For this purpose, the calculation is based on the conservation equation of energy.

(14) $$\rho \cdot c_{p}\cdot \left(\frac{\partial T}{\partial t}+\vec{v}\cdot \nabla T\right)=\lambda \cdot \nabla^{2}T+\tau \cdot \nabla \vec{v})$$

In differential notation and with Cartesian coordinates, Equation (15) results for a flow in the screw channel. Here the term left of the equals sign corresponds to the change of the internal energy. The first term after the equals sign represents the change in energy due to heat conduction. The second term indicates the reversible part of the work in the form of compression work, while terms three and four describe the non-reversible part of work by dissipation.

(15) $$\begin{array}{ll} \rho \cdot c_{p}\cdot & \left(\frac{\partial T}{\partial t}+v_{x}\cdot \frac{\partial T}{\partial x}+v_{y}\cdot \frac{\partial T}{\partial y}+v_{z}\cdot \frac{\partial T}{\partial z}\right)\\ & \;=-\left(\frac{\partial {\dot{q}}_{x}}{\partial x}+\frac{\partial {\dot{q}}_{y}}{\partial y}+\frac{\partial {\dot{q}}_{z}}{\partial z}\right)-T\cdot {\left(\frac{\partial p}{\partial T}\right)}_{p}\cdot \left(\frac{\partial v_{x}}{\partial x}+\frac{\partial v_{y}}{\partial y}+\frac{\partial v_{z}}{\partial z}\right)\\ & \;-\left(\tau_{\mathrm{xx}}\cdot \frac{\partial v_{x}}{\partial x}+\tau_{\mathrm{yy}}\cdot \frac{\partial v_{y}}{\partial y}+\tau_{\mathrm{zz}}\cdot \frac{\partial v_{z}}{\partial z}\right)\\ & \;-\left[\tau_{\mathrm{xy}}\cdot \left(\frac{\partial v_{x}}{\partial y}+\frac{\partial v_{y}}{\partial x}\right)+\tau_{\mathrm{xz}}\cdot \left(\frac{\partial v_{x}}{\partial z}+\frac{\partial v_{z}}{\partial x}\right)+\tau_{\mathrm{yz}}\cdot \left(\frac{\partial v_{y}}{\partial z}+\frac{\partial v_{z}}{\partial y}\right)\right] \end{array}$$

By the following simplifications, the energy conservation equation can be reduced and thus greatly simplified [Lak15]:

1. Constant material parameters in the considered calculation section.
2. Applying kinematic reversal.
3. Incompressible melt → $c_{v}=c_{p}$.
4. Stationary flow, therefore no time-dependent changes → $\frac{\partial T}{\partial t}=0$.
5. Melt channel completely filled with wall-adhering melt.
6. Velocity components only in channel length direction z → $v_{x}=v_{y}=0$.
7. Neglecting the heat flow in the channel length and channel width direction, temperature gradient is formed only in channel height direction → ${\dot{q}}_{x}={\dot{q}}_{z}=0$.
8. Channel height significantly smaller than channel width, so influence of flight negligible. Due to purely viscous material, normal stresses negligible → $\tau_{\mathrm{xx}}=\tau_{\mathrm{yy}}=\tau_{\mathrm{zz}}=0$.
9. Consideration of Fourier law of heat conduction → ${\dot{q}}_{y}=-\lambda \cdot \frac{\partial T}{\partial y}$.

The simplifications and assumptions result in the following relationship:

(16) $$\rho \cdot c_{p}\cdot v_{z}\cdot \frac{\partial T}{\partial z}=-\lambda \cdot \frac{\partial^{2}T}{\partial y^{2}}-\tau_{\mathrm{yz}}\cdot \left(\frac{\partial v_{z}}{\partial y}\right)$$

The dissipative energy can be described by the flow behavior of plastics and the shear rate in the screw channel. The mean shear rate is defined as follows:

(17) $$\frac{\partial v_{z}}{\partial y}=\bar{\dot{\gamma }}=\frac{v_{0}}{h}$$

The shear stress in the channel can be covered by the power law for polymer melts.

(18) $$\tau =K\left(T\right)\cdot {\dot{\gamma }}^{n}=K\left(T_{0}\right)\cdot e^{-\beta *\left(T-T_{0}\right)}\cdot {\dot{\gamma }}^{n}$$

where K(T_0_) describes the consistency factor at temperature (T_0_); β is a parameter for the temperature dependence of viscosity which has been determined empirically from measurements. Furthermore, the equation can be simplified by using the dimensionless ratios Graetz and Brinkmann. Here, the Graetz number describes the ratio of convective heat transport in the channel length direction to heat conduction in the channel height direction. The Brinkmann number, on the other hand, describes the input of dissipative energy in the screw channel compared to heat conduction in the channel height direction. Furthermore, the dimensionless coordinates ξ are introduced as dimensionless height, ζ as dimensionless channel length and Θ as dimensionless temperature.

(19) $$\mathrm{Gz}=\frac{c_{p,m}\cdot \dot{m}\cdot h}{\lambda_{m}\cdot b\cdot \Delta z}$$

(20) $$\mathrm{Br}=\frac{K\cdot v_{0}^{1+n}\cdot h^{1-n}}{\lambda_{m}\cdot T_{B}}$$

(21) $$\xi =\frac{y}{h}$$

(22) $$\zeta =\frac{z}{\Delta z}$$

(23) $$\Theta =\frac{T-T_{0}}{T_{B}}$$

(24) $$\frac{\partial \Theta }{\partial \zeta }=\frac{1}{\mathrm{Gz}}\cdot \frac{\partial^{2}\Theta }{\partial \xi^{2}}+\frac{\mathrm{Br}}{\mathrm{Gz}}\cdot \exp\left[-\beta \cdot \left(T_{B}\cdot \Theta \right)\right]$$

The simplified energy conservation Equation (24) cannot be solved analytically in a closed way due to the exponential term and must therefore be linearized. This results in the following mathematical relationship according to [25]:

(25) $$\exp\left[-\beta \cdot \left(T_{B}\cdot \Theta \right)\right]=\exp\left[-\beta \cdot \left(T-T_{0}\right)\right]=c_{1}-c_{2}\cdot \beta \cdot T_{B}\cdot \Theta$$

The constants obtained according to [25] and the final solution of the energy equation assuming an adiabatic screw and a constant barrel temperature are given in Appendix A.

With the aid of a third degree polynomial function (26), which describes the temperature distribution over the channel height, the temperature per calculation section on the screw can then be determined and averaged for j positions of ξ so that this temperature can be assumed for the melt eddy. This can be used at the respective screw position as the initial temperature of the melt in the disperse melting model.

(26) $$\Theta \left(\xi \right)=u_{1}\cdot \xi^{3}+u_{2}\cdot \xi^{2}+u_{3}\cdot \xi +u_{4}$$

(27) $$T_{M}=\frac{\sum_{k=1}^{j}T{\left(\xi \right)}_{k}}{j}$$

### 3.2. Disperse Melting Model

**Figure 5:**
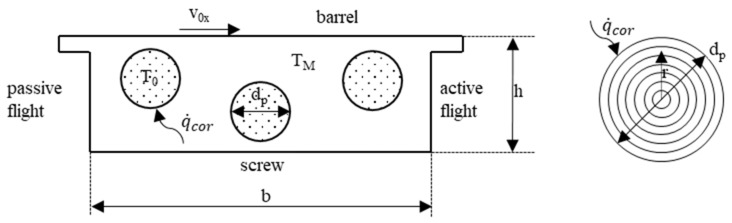
Schematic representation of disperse melting with calculation variables.

The mathematical description of disperse melting is based on the model of Pape [3], which has been partly modified and extended. In general, the model is based on the heat flow of the surrounding melt into the ideally distributed solid particles (see Figure 5). This heat flow is described by Fourier’s law of heat flow into a sphere.

By defining that the outside edge of each particle has the melting temperature T_FL_ of the respective polymer, Equation (28) can be simplified to Equation (29).

(28) $$\dot{q}=-\lambda \cdot \frac{\partial T}{\partial r}$$

(29) $$\dot{q}=-2\cdot \lambda_{m}\left(T_{M}\right)\cdot \frac{T_{M}-T_{\mathrm{FL}}}{d_{p}}$$

The respective particle radius is described with d_p_, λ_m_(T_M_) describes the thermal conductivity of the plastic at melt temperature. As previously mentioned, convection also has a considerable influence on the heat flow into the solid particle. Due to this fact, Pape [3] has introduced correction factors which take convection (factor f_k_) and the influence of particle size (factor f_lh_) on the heat flow into account. These correction factors were obtained by a regression model from numerous CFD simulations. They are defined as follows:

(30) $$f_{k}=1+\frac{\left(\left(0.0145+\left(\frac{7}{20}\right)\cdot \left(\frac{d_{p}}{h}\right)-0.655\cdot \left({\left(\frac{d_{p}}{h}\right)}^{2}\right)+\left(\frac{1}{3}\right)\cdot \left({\left(\frac{d_{p}}{h}\right)}^{3}\right)\right)\right)\cdot \sqrt{\frac{\rho_{m}\left(T_{M}\right)\cdot c_{p}\left(T_{M}\right)\cdot v_{0z}}{\lambda_{m}\left(T_{M}\right)\cdot d_{p}}}}{1+\left(\left(0.115\cdot \left(\frac{d_{p}}{h}\right)\right)-\left(\frac{1}{8}\right)\cdot \left({\left(\frac{d_{p}}{h}\right)}^{3}\right)\right)\cdot \sqrt{\frac{\rho_{m}\left(T_{M}\right)\cdot c_{p}\left(T_{M}\right)\cdot v_{0z}}{\lambda_{m}\left(T_{M}\right)\cdot d_{p}}\;}}$$

(31) $$f_{\mathrm{lh}}=1+0.25\cdot \left[\left(d_{p}/h\right)\cdot \log\left(\frac{\left(\frac{1}{{\left(d_{p}/h\right)}^{2}}\right)+1}{{\left(\frac{1-d_{p}/h}{d_{p}/h}\right)}^{2}}\right)+\frac{\pi }{2}-\mathrm{atan}\;\left(\frac{\left(\frac{1}{{\left(d_{p}/h\right)}^{2}}\right)-1}{\left(\frac{2}{d_{p}/h}\right)}\right)\right]$$

(32) $${\dot{q}}_{\mathrm{cor}}=-2\cdot \lambda_{m}\left(T_{M}\right)\cdot \frac{T_{M}-T_{\mathrm{FL}}}{d_{p}}\cdot f_{k}\cdot f_{\mathrm{lh}}$$

These factors allow one to determine a corrected heat flow into the particle, depending on melt temperature, convection and the particle size in relation to the channel depth. This heat flow leads to an increasing temperature in the solid particle, until it finally melts.

## 4. Novel Disperse Melting Model

In the following, the novel disperse melting model developed in this work is presented. It is based on the Model of Pape [3], but also covers a more detailed description of the temperature profile in the solid particles. Furthermore, the temperature development in the melt due to (i) shear increase, (ii) cooling effects of the particles and (iii) the different initial temperatures of the particles because of the changing solid bed temperature is taken into account.

### 4.1. Temperature Profile in Particle and Melting of Particle

The heat flow determined in Equation (32) of the Model of Pape [3] is used to calculate the disperse melting in this work. This heat flow leads to the formation of a temperature profile within the solid particle. This phenomenon can be regarded as a transient heat conduction problem. Thus, the following differential equation describes the temperature profile:

(33) $$\frac{\partial T}{\partial t}=\frac{\lambda_{s}}{\rho_{s}\cdot c_{p,s}}\cdot \left(\frac{\partial^{2}T}{\partial r^{2}}+\frac{2}{r}\cdot \frac{\partial T}{\partial r}\right)$$

This differential equation can be transformed into an analytically solvable equation by means of an infinite series. According to Carslaw and Jaeger [26], the following radial temperature profile is obtained at calculation interval i in the particle:

(34) $$T_{i}\left(r\right)=\frac{-3\cdot {\dot{q}}_{\mathrm{cor}}\cdot t}{\rho_{s}\cdot c_{p}\left(T_{i-1}\right)\cdot r}+\frac{-{\dot{q}}_{\mathrm{cor}}\cdot \left(5R^{2}-3r^{2}\right)}{10\cdot \lambda_{s}\cdot r}-[\frac{-2\cdot {\dot{q}}_{\mathrm{cor}}\cdot r^{2}}{\lambda_{s}\cdot R}\cdot \left(\sum_{n=1}^{\infty }\frac{\sin\left(r\cdot \frac{\mu_{n}}{r}\right)}{\mu_{n}^{2}\cdot \sin\left(\mu_{n}\right)}\cdot e^{-a\cdot \mu_{n}^{2}\cdot \frac{t}{r^{2}}}\right)]+T_{i-1}\left(r\right)$$

This makes it possible to determine the temperature in the particle independent of the residence time t in the melt, the material properties and the existing temperature T_i__−1_ of the particle. Here again, an iterative procedure is to be chosen. In addition to dividing the screw into individual sections, it is also recommended to divide the particle into several layers. This is due to the fact that the particle diameter changes as soon as one layer reaches the melting temperature. This layer changes into melt, the particle radius and also the effective surface of the particle on which the previously defined heat flow acts changes. The residence time t in the respective calculation sections can be determined by the mass or volume flow and the respective free volume of the screw channel.

(35) $$t=\frac{\dot{V}}{V_{\mathrm{channel}}}=\frac{\dot{m}}{\rho_{m}\left(T_{M}\right)\cdot V_{\mathrm{channel}}}=\frac{\dot{m}}{\rho_{m}\left(T_{M}\right)\cdot b\cdot h\cdot \Delta z}$$

It should also be noted that in Equation (34), the specific heat capacity c_p_ of the plastic is taken at a specific temperature T. This is unproblematic for materials with relatively unchangeable heat capacities above temperature, but semi-crystalline plastics have very variable specific heat capacities in the melting range. If only the value of the heat capacity at temperature T is chosen, it is possible that the temperature increase is greatly overestimated. In the following, a typical heat capacity profile and the resulting enthalpy profile of a semi-crystalline thermoplastic can be seen. Because of the peak in the heat capacity at the melting temperature of the polymer, more energy is required to heat up the polymer. This can also be seen in the slightly stronger increase of the enthalpy in the same temperature range. The increased required energy cannot be taken into account by means of a local, unchangeable heat capacity used in Equation (34).

**Figure 6:**
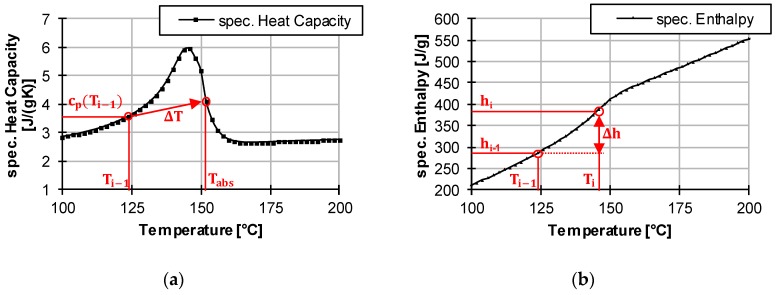
Comparison of the temperature difference when considering the specific heat capacity (**a**) or the specific enthalpy (**b**).

This is illustrated by an example: If one assumes that the particle has a temperature of 124 °C and weighs 1 g, and that the energy introduced is 100 J, a temperature rise to 151.77 °C is calculated (Figure 6a).

(36) $$\Delta T=\frac{E_{\mathrm{input}}}{m\cdot c_{p}\left(T_{i-1}\right)}=\frac{100\;J}{1\;g\;\cdot 3.6\;\frac{J}{g*K}}=27.77\;K\;\to T_{\mathrm{abs}}=151.77\;{}^{\circ}C$$

However, if one now considers the enthalpy input, the difference becomes clear. By adding 100 J/1g to the specific enthalpy h_i__−1_ at 124 °C, a specific enthalpy h_i_ of 386.57 J/g at interval i is reached (see Figure 6b). This results in a temperature of only 145.7°C instead of the temperature of 151.7 °C calculated purely by heat capacity. Thus, the temperature change in the particle must be corrected in each case over the enthalpy calculation.

(37) $$h_{i-1}=h\left(124\;{}^{\circ}C\right)=286.57\;\frac{J}{g}\to h_{i}=h_{i-1}+\frac{E_{\mathrm{input}}}{m}=286.57\frac{J}{g}+\frac{100\;J}{1\;g}=386,57\frac{J}{g}=h\left(145.7\;{}^{\circ}C\right)$$

**Figure 7:**
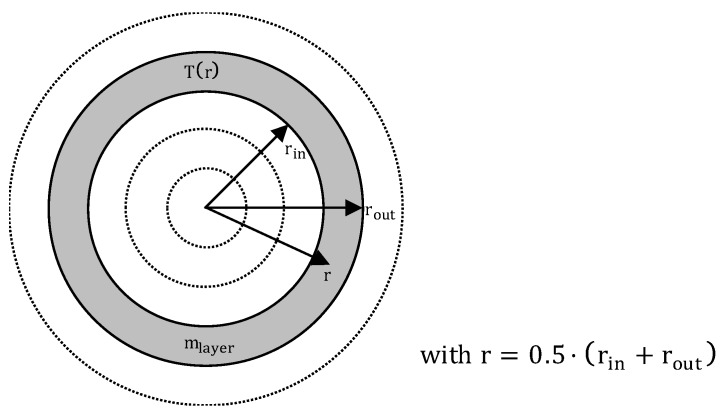
Schematic representation of different layers of the particle and the corresponding symbols.

This can be done by a short calculation step. Therefore, the temperature increase of Equation (34) at interval i is used. With this temperature increase, the introduced energy can be calculated. At the start of the calculation, the particle is divided into 100 layers with an inner radius r_in_ and an outer radius r_out_ (see Figure 7).

For each layer, the respective temperature T_i_(r) is taken into account. The introduced energy can be calculated with the following equation by converting Equation (36) to E_input_.

(38) $$E_{\mathrm{input}}=\left(T_{i}-T_{i-1}\right)\cdot m_{\mathrm{layer}}\cdot c_{p}\left(T_{i-1}\right)=\left(T_{i}-T_{i-1}\right)\cdot \frac{4}{3}\cdot \left(r_{\mathrm{out}}^{3}-r_{\mathrm{in}}^{3}\right)\cdot \pi \cdot \rho_{s}\cdot c_{p}\left(T_{i-1}\right)$$

This calculated energy input can now be added to the specific enthalpy of the last calculation step. The new specific enthalpy of the actual calculation step can then be calculated as follows:

(39) $$h_{i}=h_{i-1}+\frac{E_{\mathrm{input}}}{m_{\mathrm{layer}}}\to T\left(h_{i}\right)=T_{i}\left(r\right)$$

Subsequently, the temperature T(h_i_) associated with the enthalpy h_i_ can be taken from the measurement of the material data of the polymer and set as the corrected layers temperature T_i_(r).

### 4.2. Temperature Development of the Melt

When considering Equation (29), it is noticeable that the temperature of the melt correlates directly with the heat flow acting on the particle. Thus, the correct description of the melt temperature is of high relevance. The temperature of the melt is mainly influenced by three factors: (i) the barrel wall temperature, (ii) the resulting dissipation and (iii) the cooling of the melt by the melting particles. The latter will be dealt with first.

Due to the heat flow of melt into the solid particles, the temperature of the melt is lowered. This is a positive effect of disperse melting, as the melt temperature can be lowered, and still, a fast and homogeneous melting can be guaranteed. In order to describe the temperature reduction, it is first necessary to determine an absolute heat flow, which flows from melt into the respective particle. To do this, the surface area of a particle is determined and multiplied by the specific heat flow from Equation (32). This can subsequently be multiplied by the number of particles to calculate a total absolute heat flow from melt into all particles in the section. The number of all particles present in the section can be determined from the known solids content and the channel volume.

(40) $${\dot{Q}}_{\mathrm{cor}}={\dot{q}}_{\mathrm{cor}}\cdot A_{\mathrm{particle}}={\dot{q}}_{\mathrm{cor}}\cdot \left(\pi \cdot d_{p}^{2}\right)$$

(41) $${\dot{Q}}_{\mathrm{total}}={\dot{Q}}_{\mathrm{cor}}\cdot n_{\mathrm{particle}}$$

(42) $$n_{\mathrm{particle}}=\frac{V_{\mathrm{channel}}\cdot \psi_{v}}{V_{\mathrm{particle}}}$$

Using the total heat flow and the residence time per calculation interval, the cooling energy E_cool_ is calculated. By relating this cooling energy to the existing mass of melt multiplied by the heat capacity of the melt, an absolute temperature change ΔT_cool_ is obtained. For further simplification, the mass of the existing melt can be described by the channel geometry, the melt density and the respective solid volume content.

(43) $${\Delta T}_{\mathrm{cool}}=\frac{E_{\mathrm{cool}}}{c_{p,m}\left(T_{M}\right)\cdot m_{m}}=\frac{E_{\mathrm{cool}}}{c_{p,m}\left(T_{M}\right)\cdot V_{m}\cdot \rho_{m}\left(T_{M}\right)}=\frac{{\dot{Q}}_{\mathrm{total}}\cdot t}{c_{p,m}\left(T_{M}\right)\cdot \left(b\cdot h\cdot \Delta z\right)\cdot \left(1-\psi_{v}\right)\cdot \rho_{m}\left(T_{M}\right)}$$

In addition to the cooling of the melt by the heat flow into the solid particles, dissipation and heat exchange with the barrel wall also occur. Equations (19)–(27) can be used to determine the temperature difference. While the Graetz number according to [25] can also be used unchanged for disperse melting, a modification is necessary for the Brinkmann number.

(44) $$\mathrm{Gz}=\frac{c_{p,m}\left(T_{M}\right)\cdot \dot{m}\cdot h}{\lambda_{m}\left(T_{M}\right)\cdot b\cdot \Delta z}$$

(45) $$\mathrm{Br}=\frac{K_{\mathrm{cor}}\cdot v_{0}^{1+n}\cdot h^{1-n}}{\lambda_{m}\left(T_{M}\right)\cdot T_{B}}$$

In the Brinkmann number, the consistency factor K is used. However, in the case of disperse melting, this cannot be assumed to be merely a consistency factor of the plastic melt. Rather, various publications [27,28,29,30] have shown that the solid particles distributed in the melt lead to an increase in shear, resulting in increased dissipation. This is described by a corrected consistency factor. The reason for the shear increase is that no shear occurs within the solid particle. This reduces the effective height at which the velocity gradient and thus the shear occurs. This is illustrated as a simplified example in the following figure. The solid particles are shown here as bars of thickness d_p_. This reduces the height h available for shearing to h − d_p_·n_particle_. The shear therefore increases.

(46) $$\dot{\gamma }=\frac{v_{0z}}{h}{\;\mathrm{with}\;\Psi }_{v}=0$$

(47) $$\dot{\gamma }=\frac{v_{0z}}{h-d_{p}\cdot n_{\mathrm{particle}}}{\;\mathrm{with}\;\Psi }_{v}\neq 0$$

**Figure 8:**
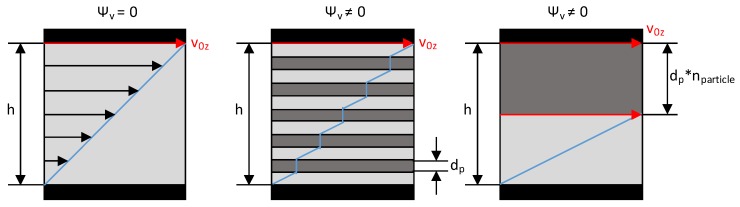
Schematic simplified representation of shear increase due to solid particles.

The modeling shown in Figure 8 is strongly simplified and only meant to visualize the phenomena of shear increase. In the extrusion process, further influences, such as cross-sectional flow, particle movement within the melt and changing temperatures of the melt lead to a more complex shear increase. Therefore, different existing models of shear increase—Batchelor, Krieger and Dougherty and Pape—were investigated in this work for their ability to represent the behavior of solid particles in the melt. For this purpose, a LDPE (LyondellBasell Lupolen 1840D) and a PP (Borealis RD204 CF) were compounded with different amounts of fine glass beads. These mixtures were subsequently measured at the high-pressure capillary rheometer (HCR) and compared with the viscosity of the pure polymer. The glass beads represent solid particles in the melt. The very high melting point of the glass beads ensures that there is no change in the solid content during the measurement. In the literature, the increase in viscosity is commonly expressed in terms of relative viscosity. This describes the ratio of the viscosity of a mixture of melt and solids to the viscosity of the pure melt.

(48) $$\eta_{\mathrm{rel}}=\frac{\eta_{\Psi_{v}}}{\eta_{0}}$$

(49) $$\mathrm{BATCHELOR}:\;\eta_{\mathrm{rel}}=1+2.5\cdot \Psi_{v}+6.2\cdot \Psi_{v}$$

(50) $$\mathrm{KRIEGER}\;\mathrm{AND}\;\mathrm{DOUGHERTY}:\;\eta_{\mathrm{rel}}={\left(1-\frac{\Psi_{v}}{\Psi_{v\max}}\right)}^{-2.5\cdot \Psi_{v\max}}\;\mathrm{with}\;\Psi_{v}{}_{\max}=0.74048$$

(51) $$\mathrm{PAPE}:\;\eta_{\mathrm{rel}}=\left(1+2.5\cdot \Psi_{v}\right)\cdot f_{\mathrm{dh}}$$

(52) $$f_{\text{dh}}=1+\psi_{v}\cdot \frac{\frac{d_{P}}{h}\cdot \left[1+{\left(\frac{d_{P}}{h}\right)}^{2}\right]}{\left[{\left(\frac{d_{P}}{h}\right)}^{2}+\frac{d_{P}}{h}+4\right]\cdot \left(1-\frac{d_{P}}{h}\right)}$$

**Figure 9:**
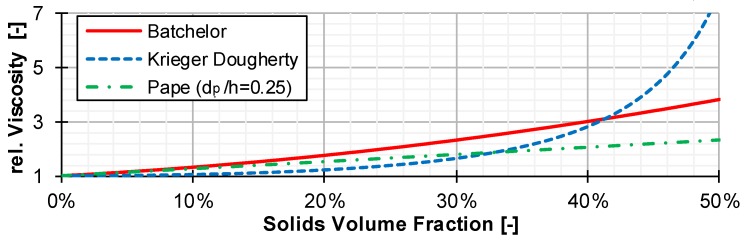
Comparison of the relative viscosities of different models over the solids contents.

**Figure 10:**
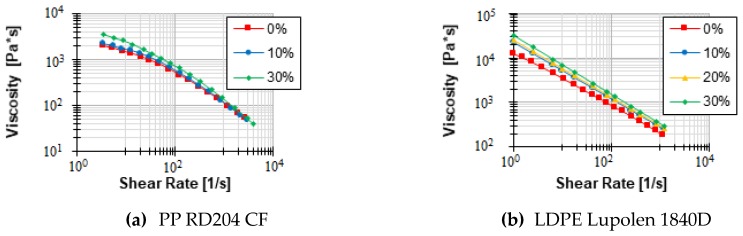
Viscosity above the shear rate for PP (**a**) and LDPE (**b**) and various solid contents.

The models described above all react very differently to rising solid contents (see Figure 9). To generate a representative ratio of particle diameter to channel depth, glass beads with diameters of 250 μm were used. The slit capillary used at the HCR had a thickness of 1 mm. Thus, a typical d_p_/h ratio of 1/4 is represented. The investigations all showed that the insertion of solid particles into the melt merely shifts the viscosity level towards higher viscosities. The flow exponent only varies within the limits of the measurement inaccuracies. The measured viscosities are shown in Figure 10.

**Figure 11:**
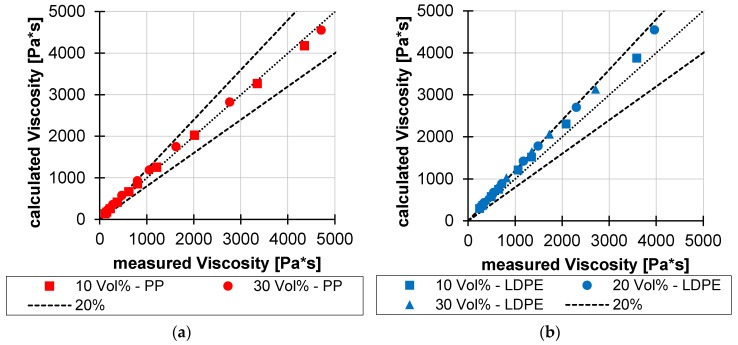
Comparison of measured and calculated viscosities of PP (**a**) and LDPE (**b**).

The models of Batchelor and Krieger and Dougherty clearly overestimate the viscosity at higher solids contents. However, this range is particularly relevant for disperse melting, since disperse melting is often initiated here. The model of Pape, on the other hand, matches the viscosities with an average deviation of 13% (PP) and 14% (LDPE) very well (see Figure 11), so that they can be used for the calculation of the corrected consistency factor.

The corrected Brinkmann number can thus be determined as follows:

(53) $$\mathrm{Br}=\frac{K_{\mathrm{cor}}\cdot v_{0}^{1+n}\cdot h^{1-n}}{\lambda_{m}\left(T_{M}\right)\cdot T_{B}}=\frac{K\left(T_{\mathrm{FL}}\right)\cdot \left(\left(1+2.5\cdot \Psi_{v}\right)\cdot f_{\mathrm{dh}}\right)\cdot v_{0}^{1+n}\cdot h^{1-n}}{\lambda_{m}\left(T_{M}\right)\cdot T_{B}}$$

According to Equations (19)–(27), the temperature increase ΔT_Dis_ due to dissipation and heat conduction of the barrel wall can then be determined.

(54) $$\Theta_{\mathrm{Dis}}\left(\xi \right)=\frac{T_{\mathrm{Dis}}\left(\xi \right)-T_{M_{i-1}}\left(\xi \right)}{T_{B}}=f\left(\beta ,{\;T}_{B},T_{M_{i-1}},\xi ,\mathrm{Br},\mathrm{Gz},\ldots \right)$$

(55) $$T_{M_{\mathrm{Dis}}}=\frac{\sum_{k=1}^{j}T_{\mathrm{Dis}}{\left(\xi \right)}_{k}}{j}$$

(56) $${\Delta T}_{\mathrm{Dis}}=T_{M_{\mathrm{Dis}}}-T_{M}{}_{i-1}$$

The resulting temperature of the melt surrounding the particles in interval i is finally formed by the sum of the temperature differences:

(57) $$T_{M_{i}}=T_{M_{i-1}}+{\Delta T}_{\mathrm{cool}}+{\Delta T}_{\mathrm{Dis}}$$

### 4.3. Determination of the Particle Temperature at the Beginning of Disperse Melting

The initial temperature of the particle is very relevant for the correct determination of the melting behavior of the disperse melting. It has a decisive influence on how much energy must be supplied to the particle by heat conduction from the melt until it gradually melts. A high initial temperature, therefore, favors disperse melting. Since the disperse melting is usually preceded by conventional melting in single-screw extrusion, the energy input into the solid is of special relevance.

Due to the fact that in conventional melting the solid is present as a solid bed, the temperature development in the solid bed is of interest for determining the initial temperature. This solid bed is broken up during disperse melting, so that the heated particles are distributed in the melt. There are already various approaches for determining the temperature development in the solids bed. The approach of Rauwendaal [31] should be mentioned in particular. It considers heat conduction purely in the direction of the channel height. An approximation is presented here which describes the temperature above the channel height.

(58) $$T\left(y\right)=\left(T_{M}-T_{\mathrm{start}}\right)\cdot \exp\left(\frac{y\cdot v_{\mathrm{sy}}}{a_{s}}\right)+T_{\mathrm{start}}$$

It should be noted here that it is assumed that only a very thin layer of the solid bed heats up and the rest of the material remains at its initial temperature T_0_. However, this only applies to a solid bed with a thickness of more than 10 mm at residence times of less than 1–2 min [31]. Especially with small screw diameters, this thickness is often lower, so that the equation is no longer applicable. A new model was developed to calculate a meaningful temperature distribution in the solids bed. This model is based on the two-dimensional finite difference method (FDM). It is also assumed that the solid bed increases in temperature purely through heat conduction. The temperature in the melt film T_film_ and the temperature of the melt in the melt eddy T_melt_ are considered as boundary conditions. The screw base and the screw flights are assumed to be adiabatic. The calculation grid is also selected equidistant, so that a fast iterative calculation can be performed. Furthermore, the melt film thickness is assumed to be the averaged melt film thickness $\bar{\delta }$

(59) $$\bar{\delta }=\frac{\delta_{0}}{1+c}$$

**Figure 12:**
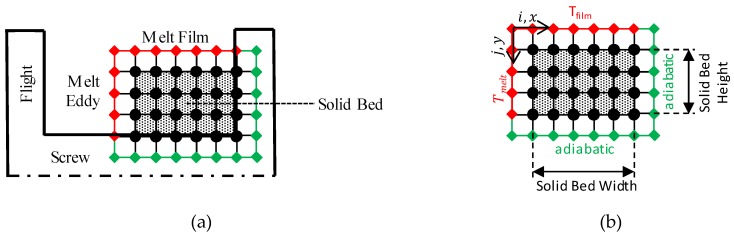
Representation of calculation grid for determination of solid bed temperature at (**a**) the screw and (**b**) in detail.

The following Figure 12 illustrates the procedure.

The differential equation of the present problem is as follows:

(60) $$\frac{\partial T}{\partial t}=a\cdot \left(\frac{\partial^{2}T}{\partial x^{2}}+\frac{\partial^{2}T}{\partial y^{2}}\right)\;\mathrm{and}\;\Delta x=\Delta y$$

Using the finite-difference method for a two-dimensional problem and the Fourier number, this can be converted into a difference equation:

(61) $$\mathrm{Fo}=a_{s}\cdot \frac{t}{\Delta x^{2}}$$

(62) $$T_{1_{i,j}}=\mathrm{Fo}\cdot \left(T_{0_{i-1,j}}+T_{0_{i+1,j}}+T_{0_{i,j-1}}+T_{0_{i,j+1}}-4\cdot T_{0_{i,j}}\right)+T_{0_{i,j}}$$

The critical time step t must be observed. This must not be chosen too large in order to guarantee a stable calculation method. Based on this fact, a maximum time step is defined. This is based on the convergence criterion according to [32]. However, since this is defined for the one-dimensional case, it must be halved again for the two-dimensional case [33]. As soon as the residence time in a calculation interval is above the critical time step, the actual calculation is divided into several calculation steps so that the actual residence time is finally reached.

(63) $$t_{\max_{1}D}=\frac{1}{2}\cdot \frac{{\Delta x}^{2}}{a_{s}};{\;t}_{\max_{2}D}=\frac{1}{4}\cdot \frac{{\Delta x}^{2}}{a_{s}}$$

**Figure 13:**
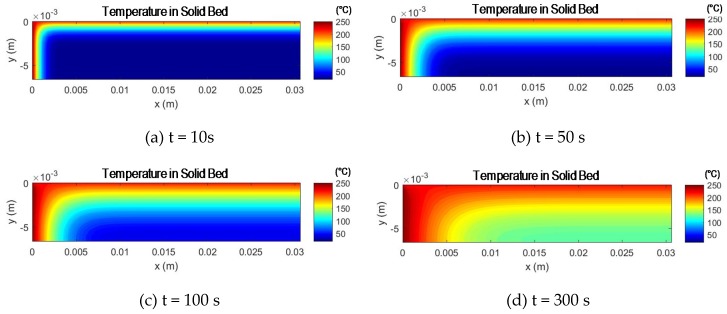
Temperature of solid bed at D = 45 mm extruder metering section at different residence times (**a**–**d**). Width = 30 mm, height = 6 mm, T_start_ = 20 °C, T_melt_ = 250 °C, T_film_ = 220°C, material: PP RD204CF.

**Figure 14:**
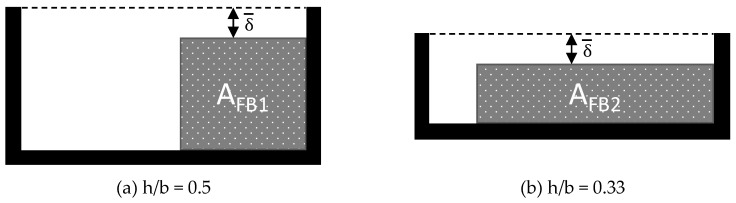
Comparison of the solid bed geometries with constant solids contents but variable channel depths.

At the beginning of the calculation process, the solid bed temperature is set to constant T_0_, which corresponds to the inlet temperature of the granulate into the extruder. Using the residence time per calculation segment and the known temperatures of the melt film and melt in the melt eddy, the temperature can then be determined at each grid point T_i,j_. An exemplary process is shown in the following Figure 13:

It can be seen that even extruders with a size of D = 45 mm and a residence time of less than 2 min can cause a significant temperature increase in the solid bed. In addition, it is not yet considered that a change in the width and height of the solids bed can be caused not only by melting, but also by possible changes in the channel’s cross-section. Above all, compression of the solids bed can lead to a faster increase in temperature in the solids bed, since the height of the solids bed is reduced (see Figure 14, A_FB1_ = A_FB2_ with a constant solids content) and the heat from the melt film reaches deeper into the solids bed. 

**Figure 15:**
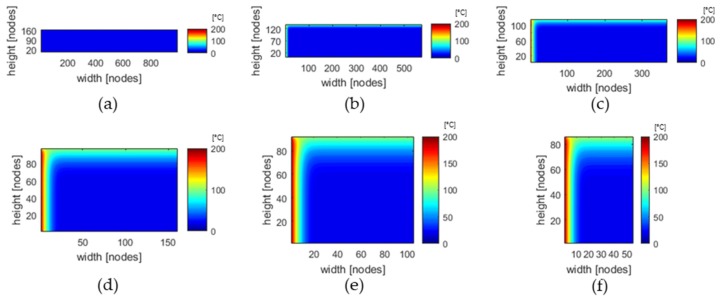
Temperature of solid bed of an D = 60 mm extruder with a three-section screw at axial position (**a**) 9 D, (**b**) 13.5 D, (**c**) 16 D, (**d**) 18.2 D, (**e**) 18.8 D, (**f**) 19.5 D. The nodes are equidistant for (**a**)–(**e**), starting with 1000 nodes for the solid bed at the beginning of the screw.

To take the deformation into account, the geometry of the solid bed is compared in each calculation step with the geometry from the previous calculation step. If the width becomes smaller, melting can be assumed. The nodes of the FDM model are thus considered as melted material and are not considered further. If the height of the solid bed decreases, compression is assumed. The equidistant grid is retained, but the temperatures at the nodes are interpolated in the channel height direction using the temperatures of the nodes of the previous calculation segment, so that the same temperature profile is obtained at the reduced height. If there is a widening of the solid bed, this is applied accordingly for the channel width. This ensures that deformations of the solid bed do not lead to calculation errors due to nodes reaching outside the effective solid bed. A calculation of the solid bed temperature in a wave-dispersion screw would therefore lead to many chances in solid bed width and height, but is not necessary, since the wave-structure will cause the solid bed to break up and initiate disperse melting. The deforming solid bed is shown as an example in Figure 15 for a three-section screw.

To determine the mean solids bed temperature, the temperatures of all nodes of the solids bed are averaged. Due to the equidistant calculation grid, no weighting is necessary. The outside nodal points only represent the boundary conditions and therefore do not have to be considered.

(64) $$T_{\mathrm{FB}}=\frac{1}{\left(i_{\mathrm{tot}}-2\right)\cdot \left(j_{\mathrm{tot}}-2\right)}\cdot \overset{i_{\mathrm{tot}}-1}{\underset{i=2}{\sum }}\overset{j_{\mathrm{tot}}-1}{\underset{j=2}{\sum }}T_{i,j}$$

**Figure 16:**
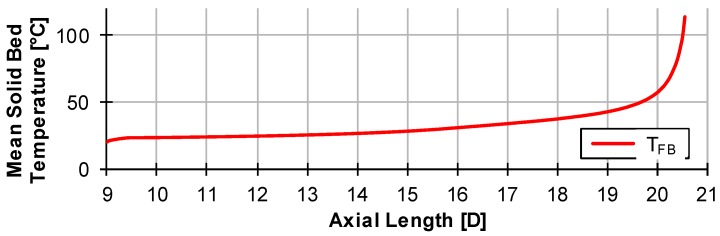
Mean Temperature of solid bed of an D = 60 mm extruder with a three-section-screw.

Here, i_tot_ and j_tot_ correspond to the number of nodes in i and j direction. The mean solid bed temperature T_FB_ can then be used as the particle initial temperature at the point of initiation of disperse melting. This requires a calculation of conventional melting according to Section 3.1, taking into account the continuous calculation of the solid bed temperature. Thus, all necessary mathematical correlations and boundary conditions are defined in order to be able to perform a calculation of disperse melting. The mean temperature of the solid bed of the example in Figure 15 is shown in Figure 16. Here, the melting end of conventional melting is approximately 20.5 D.

### 4.4. Application on Wave-Dispersion Screws

**Figure 17:**
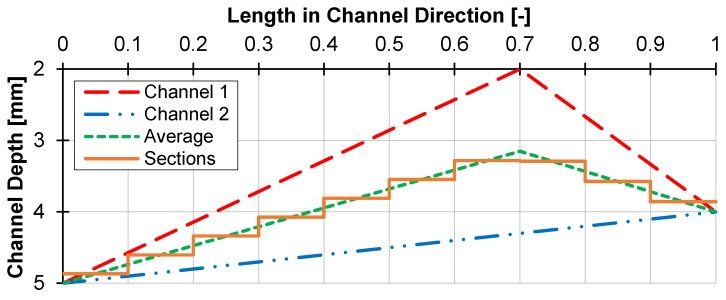
Average channel depth of a part of a wave-dispersion screw.

To apply the model to wave-dispersion screws, there are several approaches. Since the model mainly depends on the residence time and the channel geometry, it is advisable to divide the screw into many sections of constant boundary conditions and to perform the calculation for the respective sections until the entire melting behavior is calculated. Since in the work presented here, only the throughput, the thermal conditions and the screw geometry are given as boundary conditions, and thus it is not possible to differentiate between different throughputs in the two screw channels, the channel height between the two channels of the wave-dispersion screw is averaged. The width of the channel is then selected such that the resulting channel cross section corresponds to the sum of the channel cross sections of channel 1 and channel 2. Thus, the calculation can be started only by specifying the throughput, the thermal boundary conditions and the screw geometry, which allows for easy adaptation to a wide variety of calculation processes, even away from wave-dispersion screws. The simplification through the adapted channel width is to be classified as permissible insofar as the disperse melting is barely dependent on the channel width. Dominant here is the residence time and the shear introduced by the decreasing channel depth, which is covered by the averaged channel height. The channel width, however, is mainly relevant for the calculation of the solid bed temperature. This takes place before the disperse melting model, so that the real channel geometry data can be used there. The validation studies support this thesis. The procedure is illustrated in Figure 17. The channel depths are plotted over the length in the channel direction. In channel 1 there is a wave, while in channel 2 is only a slight compression. The mean channel depth is formed from both channel depths and then divided into exemplary 10 sections of constant boundary conditions with the average channel depth in each section, allowing one to calculate disperse melting.

A calculation of the individual channels of wave-dispersion screws requires a calculation of the pressure-throughput behavior and knowledge of the mass flows in the individual channels. This requires a coupling of the melting model with a pressure-throughput model for wave-dispersion screws, which has not yet been fully researched [14,15]. With exact quantitative knowledge of separating and combining melt flows, it would then be possible to implement weighted mixing rules for determining the melt temperature in the temperature calculation. However, since this has not been researched or even validated at the present time, the melting behavior will be calculated in the following using the averaging of the channel depths described above.

## 5. Verification

A design of experiments was carried out to verify the established model. The aim of this was to examine the effects of the individual factors on the model and to check their plausibility. In order to guarantee a simple comparability of the trial points, only metering sections with a pitch of 1 D and different channel depths were investigated. Furthermore, the diameter of the screws was varied. The simulated points are shown in Table A1 and Table A2. The resulting different channel depths and mass throughputs were calculated on the basis of the scale-up theory developed by Potente [34] using the D = 30 mm screw. This further guarantees a comparability of the individual geometries. In addition to the geometric parameters of the extruder screw, the throughput, peripheral screw speed, barrel temperature and granulate input temperature were varied on the process side. Furthermore, the start of the disperse melting process was varied between 30 and 70 vol.% solid content in order to detect an influence of the possibly increased melt cooling if the disperse melting process was initiated too early. As the last parameter to be considered, the granulate diameter was chosen variably, since smaller granulates would have to melt faster due to the changing surface to volume ratio. As material data, the data of the HDPE HE3493-LS-H were used.

**Figure 18:**
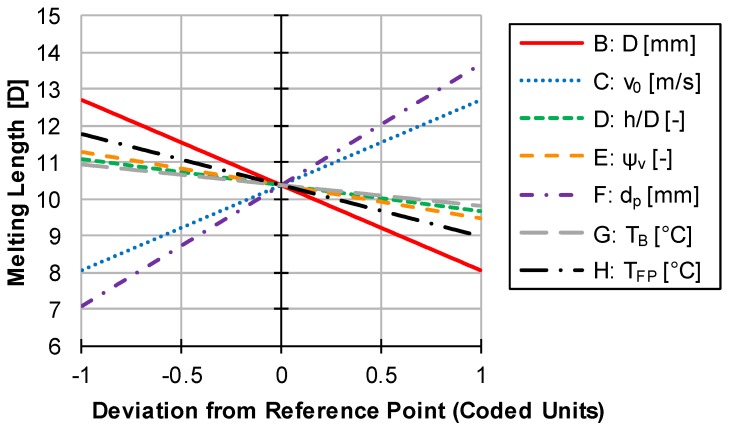
Perturbation plot of design of experiments for verification.

The program “Design Expert 12” from the company “Stat-Ease” was used to analyze the influences of the varied factors. The evaluation was done by creating a mathematical regression (see Appendix C, Table A3 and Table A4). This has a high accuracy with a corrected error square of 80.6%. The following perturbation diagram in Figure 18 can thus be considered meaningful and used for verification.

Since in the experimental design, the throughput was adjusted according to the circumferential speed of the screws and the parameters are therefore directly related, this was not considered in the evaluation. The influences of the individual factors correspond to the expectations. A larger diameter (B) of the extruder leads to a shortened melt length due to, usually, slightly increased residence times and generally more formable disperse melting in larger channels. A higher initial particle temperature (H) also shows this effect. Here, less energy has to be introduced to the particle until it finally melts, thereby shortening the melting length. The circumferential speed (C) and the particle radius (F) behave contrary. If these are increased, the melting length also increases. Higher circumferential speeds lead to higher throughputs and thus reduced residence times. This effect is stronger than the increased dissipation due to the increased shear of the melt and therefore leads to a longer melt length. Larger particles have a smaller surface to volume ratio. Thus, a smaller heat flow, related to the volume of the particles, reaches the particle; melting occurs more slowly. Small positive effects on melting have the channel height to diameter ratio (D), the solid volume content (E) and the barrel temperature (G). The latter produces a higher melt temperature and thus a higher heat flow, which accelerates melting. A greater channel depth leads to higher residence times at constant throughput and thus accelerated melting. The influence of the solid content when initiating disperse melting is of particular interest. Here it can be seen that an early initiation of disperse melting is preferable. The increased cooling of the melt by several solid particles seems to be less important than the generally increased melting capacity of disperse melting compared to conventional melting. An increased solid content also leads to a greater increase in shear and thus to greater dissipation, which counteracts the excessive cooling of the melt.

**Figure 19:**
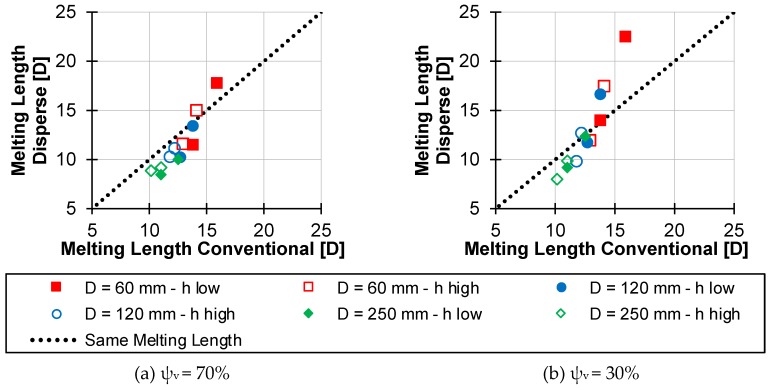
Comparison of conventional melting length and the disperse melting length for initiating disperse melting at a solid volume content of (**a**) ψ_v_ = 70% and (**b**) ψ_v_ = 30%.

The influences mentioned above are also evident when comparing the two melting models. Figure 19 shows the melting lengths of disperse melting compared with those of conventional melting. The simulation points from Table A1 of diameters 60, 120 and 250 mm are shown as examples. The simulation was performed at a barrel wall temperature of 200 °C and with the material data of the HDPE HE3493-LS-H. The inscription “h low” indicates the small channel depth, and “h high” the large channel depths of the respective diameters. Furthermore, the solid volume content at the beginning of disperse melting was differentiated into (a) ψ_v_ = 70% and (b) ψ_v_ = 30%.

Considering Figure 19, the disperse melting is advantageous if the points are below the dotted line, while conventional melting has an advantage if the points are above the dotted line. In general, it is noticeable that the majority of all points are below the dotted line. Disperse melting therefore offers a higher melting capacity, resulting in shorter melting lengths compared to conventional melting. With regard to the influences mentioned before, tendencies are also apparent. It can be seen that disperse melting has a greater advantage over conventional melting with increasing diameters. Thus, regardless of the channel depth and solid volume content, all points of the D = 250 mm simulations are below the dotted line. Furthermore, it can be seen that early initiation of disperse melting at (a) ψ_v_ = 70% results in faster melting than at (b) ψ_v_ = 30%. It should also be noted that smaller channel depths, especially with small extruders, can lead to significantly increased disperse melting lengths up to 40% higher than the conventional melting length. This is particularly evident in Figure 19b at the point in the top right-hand corner. The shallow channel depth at constant throughput leads to a short residence time. This time is not adequate for the acting heat flow to melt the particle in a reasonable time.

In summary, the disperse melting model can be regarded as verified. The occurring influences of the examined factors can all be explained physically and are also shown in comparison to the conventional melting model introduced in Section 3.1.

## 6. Validation

**Figure 20:**
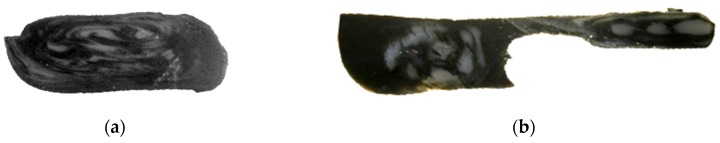
Micro sections after a wave crest (**a**) and on a wave crest (**b**).

To validate the model, experiments were carried out on various single-screw extruders using three different screw geometries of wave-dispersion screws. On the one hand, work was carried out on a D = 30 mm high-speed extruder from esde, which can reach a maximum speed of 2100 rpm. Furthermore, a D = 45 mm single-screw extruder from Battenfeld was used, which has a maximum speed of 570 rpm. An energy-transfer screw geometry on each extruder and a double-wave geometry on the D = 45 mm extruder were investigated. To determine the melting process, a cooling/pulling-out experiment (also known as Dead-Stop) was carried out at various process points. For this purpose, carbon black was added to the pure polymer granulate, the process was run stationary and then stopped abruptly. The screw with the melt was cooled down and taken out of the barrel. The solidified melt was removed from the screw channels afterwards and examined. By making thin sections of these samples across the channel, the melting behavior can be analyzed (see Figure 20).

Due to the added carbon black, the melt is black because the carbon black mixes into the melt. The solid, on the other hand, retains its original color (white in this case), as the carbon black is only deposited at the particle boundaries. Thus, an image analysis can be used to determine what percentage of the channel is black, and thus, melted, and what percentage is white, and thus, solid. The melting process can therefore be determined by taking several samples over the length of the screw.

**Figure 21:**
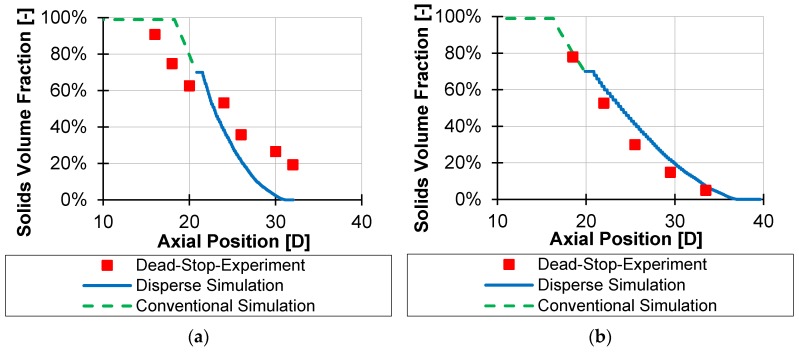
Comparison of measured versus simulated solid contents: investigation points with largest deviation (**a**) and smallest deviation (**b**).

**Figure A1:**
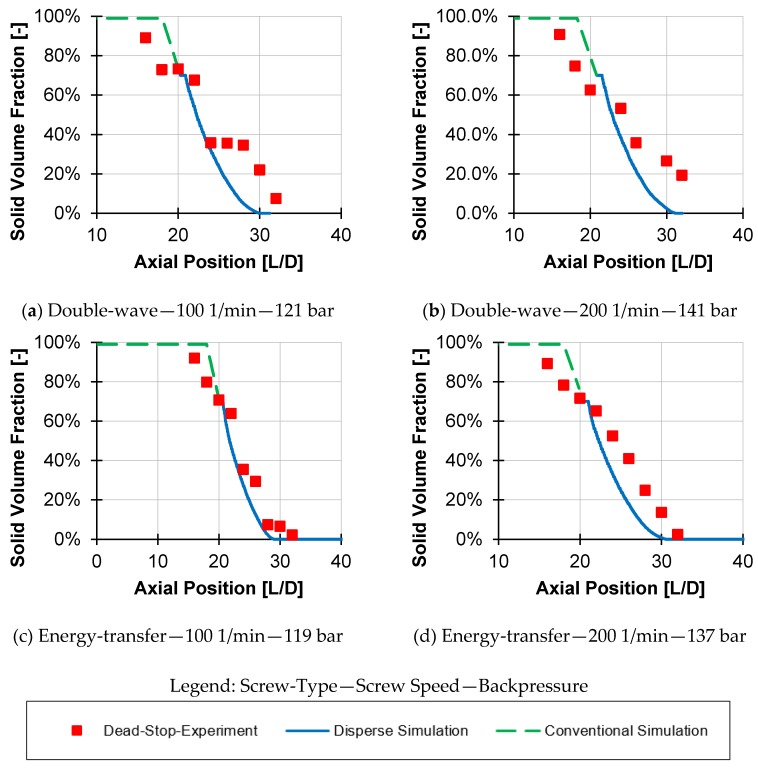
Comparison of the measured versus simulated solids contents for double-wave screw D = 45 mm (**a**,**b**) and energy-transfer screw D = 45 mm (**c**,**d**).

**Figure A2:**
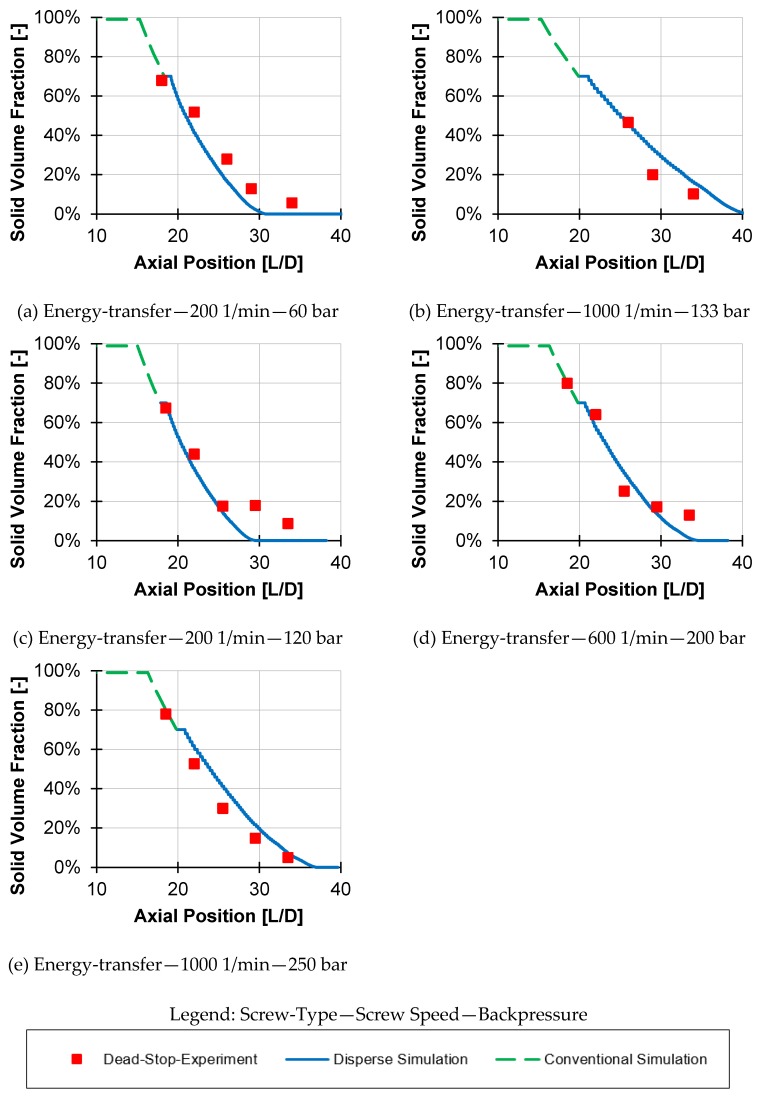
Comparison of measured versus simulated solids content for energy-transfer screw D = 30 mm with low back pressure (**a**,**b**) and high back pressure (**c**–**e**).

The investigations carried out were subsequently simulated using the melting calculations presented in Section 3 and Section 4. The start of the disperse melting process was defined as the point at which wave geometries were already present on the screw, and at the same time at least 30% of the melt was present to allow disperse melting. The complete results are given in Appendix D, Figure A1 and Figure A2. The following two results are given as examples. Figure 21 shows the point of investigation with the greatest deviation (a) and the smallest deviation (b) from the simulation. Additionally, in the processing, two points of investigation differ strongly: While (a) has a speed of only 200 rpm at a diameter of D = 45 mm and the double-wave concept, (b) has a speed of 1000 rpm at D = 30 mm using the energy-transfer concept. A higher circumferential speed and the more frequent reapplication of the energy-transfer concept leads to a more ideal disperse melting and is therefore reproduced more precisely by the model. Nevertheless, the mean deviation over all nine process points is 11% in absolute terms, and only 9% in the median. Thus, the model is suitable for estimating the melting behavior in wave-dispersion sections.

## 7. Discussion and Conclusions

By the extended consideration of the disperse melting in single screw extruders, an analytical melting model was established, which allows one to represent the disperse melting process. By considering the changeable melt temperature, taking into account the shear increase caused by the distributed solid particles and the cooling caused by the outgoing heat flow into the particles, the temperature behavior in the particle and thus the melting of the particle could be described precisely. By additionally taking into account the temperature increase in the solid bed caused by heat conduction from the surrounding melt layers, it was also possible to achieve a higher accuracy of the melting process in smaller extruders. This is also reflected in the very precise validation results of the energy-transfer screws.

In order to enlarge the validation window, further validation studies should be carried out on larger screws and with different polymers on wave-dispersion screws. Furthermore, a concept should be established regarding how non-spherical granulate particles, such as cylindrical granulate, can be covered by the disperse melting model.

In further investigations, 2-phase 3D CFD simulations can be used to investigate the complex flow processes in wave-dispersion screws in detail, and thus, possibly, to increase the accuracy of the validation points of the double-wave screws. These simulations offer the advantage that ideal spherical particles do not have to be assumed, but that by imaging the solid as a highly viscous fluid, it can be distributed in the channel according to the flow processes. The first results at Kunststofftechnik Paderborn [35], and other scientists [36,37,38], confirm the possibilities of this modeling.

## Nomenclature

*Roman Characters*	
a	temperature conductivity
$a_{s}$	temperature conductivity of solid
$A_{\mathrm{channel}}$	cross-sectional area of the channel
$A_{\mathrm{melteddy}}$	cross-sectional area of the melt eddy
$A_{\mathrm{meltfilm}}$	cross-sectional area of the melt film
$A_{\mathrm{particle}}$	surface area of particle
$A_{\mathrm{solid}}$	cross-sectional area of the solid bed
b	channel width
Br	Brinkmann number
c	contour exponent
$c_{1},c_{2}$	constants of linearization
$c_{p},c_{v}$	specific heat capacity
$c_{p,s}$	specific heat capacity of the solid
$c_{p,m}$	specific heat capacity of the melt
$c_{p}\left(T_{M}\right)$	specific heat capacity at T_M_
$d_{p}$	particle diameter
D	screw diameter
$E_{\mathrm{cool}}$	energy of cooling
$f_{k}$	correction factor of convection
$f_{\mathrm{lh}}$	correction factor of channel depth
Fo	Fourier number
Gz	Graetz number
h	channel depth
$h_{E}$	specific enthalpy
${\Delta h}_{m}$	specific melting enthalpy
${\Delta h}_{s}$	specific solid enthalpy
i, j	variable for fdm-nodes in solid bed width and height direction
$k_{1},{\;k}_{2}$	correction factors according to [16]
K	consistency factor of power law
$K_{\mathrm{cor}}$	corrected consistency factor of power law
$K\left(T_{\mathrm{FL}}\right)$	consistency factor of power law at temperature T_FL_
m	mass
$m_{m}$	mass of melt
$\dot{m}$	mass flow
${\dot{m}}_{m}$	melting mass flow
n	flow exponent of power law
$n_{\mathrm{particle}}$	number of particles
N	screw speed
p	pressure
$\dot{q}$	specific heat flow
${\dot{q}}_{\mathrm{cor}}$	corrected specific heat flow
$\dot{Q}$	heat flow
${\dot{Q}}_{\mathrm{cor}}$	corrected heat flow
${\dot{Q}}_{\mathrm{total}}$	complete corrected heat flow of all particles
r	radius of particle as control variable
R	absolute radius of the particle
t	time
$t_{\max}$	maximum time step for stability
T	temperature
${\Delta T}_{\mathrm{Dis}}$	temperature difference because of dissipation
${\Delta T}_{\mathrm{cool}}$	temperature difference because of cooling
$T_{\mathrm{FB}}$	temperature of solid bed
$T_{\mathrm{film}}$	temperature of solid bed
$T_{\mathrm{FL}}$	melting temperature
$T_{M}$	temperature of melt
$T_{\mathrm{start}}$	initial temperature of particle
$T_{B}$	temperature of barrel
$u_{1\ldots 4}$	constants for temperature polynomial
v	velocity
$v_{x},{\;v}_{y},{\;v}_{z}$	velocity in x-direction, y-direction, z-direction
$v_{0}$	circumferential speed of screw
$v_{0x},\;v_{0z}$	resulting speed in axial-direction x or channel-direction z
$v_{\mathrm{rel}}$	relative speed according to [16]
$v_{\mathrm{sy}}$	melting velocity of polymer according to [31]
$V_{\mathrm{channel}}$	volume of channel
$V_{\mathrm{particle}}$	volume of particle
$V_{m}$	volume of melt
$\dot{V}$	volume flow rate
$x_{\mathrm{FB}}$	solid bed width
Δx=Δy	distance in equidistant FDM calculation grid
z	length in channel direction
*Greek Characters*	
β	parameter for the temperature dependence of viscosity
$\dot{\gamma }$	shear rate
δ	melt film thickness at position x
$\bar{\delta }$	average melt film thickness
$\delta_{0}$	starting melt film thickness at x = b
ζ	dimensionless length
$\eta_{\mathrm{rel}}$	relative viscosity
$\eta_{0}$	viscosity of pure polymer
$\eta_{\psi_{V}}$	viscosity of polymer with $\psi_{V}$ solids
Θ	dimensionless tempature
λ	therm conductivity
$\lambda_{s}$	thermal conductivity of solid
$\lambda_{m}\left(T_{M}\right)$	thermal conductivity of melt at T_M_
$\mu_{n}$	Eigenvalue $\left(\mu_{n}=\tan(\mu_{n})\right)$
ξ	dimensionless height
ρ	density
$\rho_{m}\left(T_{M}\right)$	density at melt temperature
$\rho_{s}$	solid density
τ	shear sess
φ	pitch angle
$\psi_{v}$	solids vume fraction

## Appendix A: Final Equation to Describe the Melt Temperature in the Screw Channel According to [25]

(A1) $$\begin{array}{ll} \Theta = & \frac{\Theta_{z}\cdot \cosh\left(\sqrt{c_{2}\cdot \beta \cdot T_{B}\cdot \mathrm{Br}}\cdot \xi \right)}{\cosh\sqrt{c_{2}\cdot \beta \cdot T_{B}\cdot \mathrm{Br}}}+\frac{\Psi_{s}\cdot \sinh\left(\sqrt{c_{2}\cdot \beta \cdot T_{B}\cdot \mathrm{Br}}\cdot \left(1-\xi \right)\right)}{\sqrt{c_{2}\cdot \beta \cdot T_{B}\cdot \mathrm{Br}}\cdot \cosh\sqrt{c_{2}\cdot \beta \cdot T_{B}\cdot \mathrm{Br}}}\\ & \;+\frac{c_{1}}{c_{2}\cdot \beta \cdot T_{B}}\left[1-\frac{\cosh\left(\sqrt{c_{2}\cdot \beta \cdot T_{B}\cdot \mathrm{Br}}\cdot \xi \right)}{\cosh\sqrt{c_{2}\cdot \beta \cdot T_{B}\cdot \mathrm{Br}}}\right]\\ & \;+\sum_{n=0}^{\infty }\{e^{-\frac{\left[{\left(n+0,5\right)}^{2}\cdot \pi^{2}+c_{2}\cdot \beta \cdot T_{B}\cdot \mathrm{Br}\right]\cdot \zeta }{\mathrm{Gz}}}\\ & \;\cdot \{\cos\left(\left(n+0,5\right)\cdot \pi \cdot \xi \right)\\ & \;\cdot [-\frac{2\cdot \Theta_{z}\cdot \pi \cdot \left(n+0,5\right)}{{\left(-1\right)}^{n}\cdot \left[{\left(n+0,5\right)}^{2}\cdot \pi^{2}+c_{2}\cdot \beta \cdot T_{B}\cdot \mathrm{Br}\right]}\\ & \;-\frac{2\cdot c_{1}\cdot \mathrm{Br}}{\pi \cdot \sin\left(\left(n+0,5\right)\pi \right)\left[{\left(n+0,5\right)}^{2}\cdot \pi^{2}+c_{2}\cdot \beta \cdot T_{B}\cdot \mathrm{Br}\right]\cdot \left(n+0,5\right)}\\ & \;+\frac{2\cdot \left(u_{1}+u_{2}+u_{4}\right)}{\pi \cdot \sin\left(\left(n+0,5\right)\cdot \pi \right)\left(n+0,5\right)}-\frac{4\cdot u_{2}}{\pi^{3}\cdot \sin\left(\left(n+0,5\right)\cdot \pi \right){\left(n+0,5\right)}^{3}}]\\ & \;-\frac{2\cdot \psi_{s}\cdot \sin\left(\left(n+0,5\right)\cdot \pi \cdot \left(1-\xi \right)\right)}{\left[{\left(n+0,5\right)}^{2}\cdot \pi^{2}+c_{2}\cdot \beta \cdot T_{B}\cdot \mathrm{Br}\right]\cdot {\left(-1\right)}^{n}}\\ & \;-\frac{12\cdot u_{1}\cdot \left[\pi \cdot \left(n+0,5\right)\cdot \cos\left(\pi \cdot \left(n+0,5\right)\cdot \xi \right)-\sin\left(\pi \cdot \left(n+0,5\right)\cdot \left(1-\xi \right)\right)\right]}{\pi^{4}\cdot {\left(n+0,5\right)}^{4}\cdot \sin\left(\left(n+0,5\right)\pi \right)}\\ & \;+\frac{2\cdot u_{3}\cdot \left[\pi \cdot \left(n+0,5\right)\cdot \cos\left(\pi \cdot \left(n+0,5\right)\cdot \xi \right)-\sin\left(\pi \cdot \left(n+0,5\right)\cdot \left(1-\xi \right)\right)\right]}{\pi^{2}\cdot {\left(n+0,5\right)}^{2}\cdot \sin\left(\left(n+0,5\right)\pi \right)}\}\}\\ & \;+\frac{u_{1}\cdot \left(\xi^{3}-1\right)}{e^{\frac{c_{2}\cdot \beta \cdot T_{B}\cdot \mathrm{Br}}{\mathrm{Gz}}\cdot \zeta }}+\frac{u_{1}\cdot \left(1-\xi^{3}\right)}{e^{\frac{c_{2}\cdot \beta \cdot T_{B\cdot \mathrm{Br}}}{\mathrm{Gz}}\cdot \zeta }}+\frac{u_{2}\cdot \left(\xi^{2}-1\right)}{e^{\frac{c_{2}\cdot \beta \cdot T_{B}\cdot \mathrm{Br}}{\mathrm{Gz}}\cdot \zeta }}+\frac{2u_{2}\cdot \zeta }{\mathrm{Gz}}\cdot e^{-\frac{c_{2}\cdot \beta \cdot T_{B}\cdot \mathrm{Br}}{\mathrm{Gz}}\cdot \zeta }-u_{2}\\ & \;\cdot e^{-\frac{c_{2}\cdot \beta \cdot T_{B}\cdot \mathrm{Br}}{\mathrm{Gz}}\cdot \zeta }\cdot \left(\frac{2\cdot \zeta }{\mathrm{Gz}}+\xi^{2}-1\right) \end{array}$$

(A2) $$\Theta_{z}=\frac{T_{B}-T_{0}}{T_{B}}$$

(A3) $$c_{1}=1$$

(A4) $$c_{2}=\frac{1-\exp\left[-\beta \cdot \left(T_{B}-T_{0}\right)\right]}{\beta \cdot \left(T_{B}-T_{0}\right)}$$

## Appendix B: Simulation Points of the Design of Experiment

**Table A1:** Main simulation points of design of experiments.

No.	Throughput [kg/h]	Screw Diameter [mm]	Screw Speed [m/s]	Ch. Depth Ratio [h/D]	Initial Solid Fraction of Disperse Melting [-]
1	50	30	0.785	0.167	0.7
2	50	30	0.785	0.167	0.3
3	50	30	0.785	0.083	0.7
4	50	30	0.785	0.083	0.3
5	150	30	2.356	0.167	0.7
6	150	30	2.356	0.167	0.3
7	150	30	2.356	0.083	0.7
8	150	30	2.356	0.083	0.3
9	140	60	0.660	0.140	0.7
10	140	60	0.660	0.140	0.3
11	140	60	0.660	0.070	0.7
12	140	60	0.660	0.070	0.3
13	420	60	1.979	0.140	0.7
14	420	60	1.979	0.140	0.3
15	420	60	1.979	0.070	0.7
16	420	60	1.979	0.070	0.3
17	200	75	0.628	0.132	0.7
18	200	75	0.628	0.132	0.3
19	200	75	0.628	0.067	0.7
20	200	75	0.628	0.067	0.3
21	600	75	1.885	0.132	0.7
22	600	75	1.885	0.132	0.3
23	600	75	1.885	0.067	0.7
24	600	75	1.885	0.067	0.3
25	260	90	0.613	0.127	0.7
26	260	90	0.613	0.127	0.3
27	260	90	0.613	0.063	0.7
28	260	90	0.613	0.063	0.3
29	780	90	1.791	0.127	0.7
30	780	90	1.791	0.127	0.3
31	780	90	1.791	0.063	0.7
32	780	90	1.791	0.063	0.3
33	400	120	0.565	0.118	0.7
34	400	120	0.565	0.118	0.3
35	400	120	0.565	0.059	0.7
36	400	120	0.565	0.059	0.3
37	1200	120	1.696	0.118	0.7
38	1200	120	1.696	0.118	0.3
39	1200	120	1.696	0.059	0.7
40	1200	120	1.696	0.059	0.3
41	1200	250	0.524	0.098	0.7
42	1200	250	0.524	0.098	0.3
43	1200	250	0.524	0.049	0.7
44	1200	250	0.524	0.049	0.3
45	3600	250	1.440	0.098	0.7
46	3600	250	1.440	0.098	0.3
47	3600	250	1.440	0.049	0.7
48	3600	250	1.440	0.049	0.3

**Table A2:** Additionally varied for each of the points of Table A1 with one factor at a time method.

Level	Particle Diameter [mm]	Barrel Temperature [°C]	Particle Inlet Temperature [°C]
Low	1	180	20
Middle	3	200	50
High	5	220	80

## Appendix C: Regression from DoE for the Required Melting Length

### Regression in Coded Units

(A5) $$\begin{array}{ll} L_{{\mathrm{melt}}_{\mathrm{disperse}}}\cdot \frac{1}{D}= & 10.384-2.316\cdot B+2.318\cdot C-0.696\cdot D-0.917\cdot E+3.279\cdot F-0.563\\ & \cdot G-1.383\cdot H+0.562\cdot B\cdot E+0.817\cdot B\cdot H-0.544\cdot C\cdot E+1.135\cdot C\cdot F\\ & -0.411\cdot C\cdot H+0.589\cdot D\cdot E+0.640\cdot D\cdot F-0.382\cdot E\cdot F \end{array}$$

Adjusted Sum of Squares: 0.8061

**Table A3:** Linear coded units of 2-FI regression model.

Unit/Level	B-D [mm]	C-v_0_ [m/s]	D-h/D [-]	E-ψ_v_ [-]	F-d_p_ [mm]	G-T_B_ [°C]	H-T_FP_ [°C]
−1	30	0.524	0.049	0.3	1	180	20
0	140	1.44	0.108	0.5	3	200	50
1	250	2.356	0.167	0.7	5	220	80

### Anova

**Table A4:** Anova of modified 2-FI regression model.

Source	Sum of Squares	df	Mean Square	F-Value	p-Value
Model	4,310.983	15.000	287.399	120.491	7.20 × 10^−141^
B-D	774.333	1.000	774.333	324.636	4.61 × 10^−54^
C-v_0_	1,090.859	1.000	1,090.859	457.339	5.46 × 10^−69^
D-h/D	68.091	1.000	68.091	28.547	1.51× 10^−7^
E-ψ_v_	212.918	1.000	212.918	89.265	2.53 × 10^−19^
F-d_p_	895.785	1.000	895.785	375.555	4.37 × 10^−60^
G-T_B_	30.432	1.000	30.432	12.759	3.96 × 10^−4^
H-T_FP_	126.656	1.000	126.656	53.100	1.61 × 10^−12^
BE	45.594	1.000	45.594	19.115	1.56 × 10^−5^
BH	24.903	1.000	24.903	10.441	1.33 × 10^−3^
CE	60.160	1.000	60.160	25.222	7.60 × 10^−7^
CF	60.995	1.000	60.995	25.572	6.40 × 10^−7^
CH	7.609	1.000	7.609	3.190	7.48 × 10^−2^
DE	48.806	1.000	48.806	20.462	7.95 × 10^−6^
DF	14.876	1.000	14.876	6.237	1.29 × 10^−2^
EF	13.973	1.000	13.973	5.858	1.59 × 10^−2^

The Model F-value of 120.49 implies the model is significant. There is only a 0.01% chance that an F-value this large could occur due to noise. P-values less than 0.0500 indicate model terms are significant. In this case B, C, D, E, F, G, H, BE, BH, CE, CF, DE, DF and EF are significant model terms. Values greater than 0.1000 indicate the model terms are not significant. If there are many insignificant model terms (not counting those required to support hierarchy), model reduction may improve your model.
